# The effect of artificial aging after multi-directional forging of supersaturated AA2024

**DOI:** 10.1038/s41598-025-11453-5

**Published:** 2025-07-15

**Authors:** Mohadeseh Rashidi, Mohammad Abdian, Mohsen Kazeminezhad

**Affiliations:** Department of Materials Science and Engineering, Sharif University of Technology, Azadi Avenue, Tehran, Iran

**Keywords:** Severe plastic deformation (SPD), Multi-directional forging (MDF), AA2024, Artificial aging, Engineering, Materials science

## Abstract

The primary objective of this study is to determine the most effective solution treatment and aging temperature for AA2024 aluminum alloy to achieve superior mechanical properties. In this research, a Severe Plastic Deformation (SPD) method known as Multi-Directional Forging (MDF), which is one of the useful methods for creating Ultra-Fine Grained (UFG) microstructure, was employed on AA2024. Due to the limited studies on the effects of artificial aging on this alloy in its supersaturated state following the MDF process, the alloy was subjected to solution treatments at 480 °C, 500 °C, and 520 °C for 1 h, followed by immediate MDF. Aging was then performed at 100 °C, 140 °C, 190 °C, 240 °C, and 290 °C for 1 h each, to achieve artificial aging. To investigate the microstructure and precipitate conditions, Optical Microscopy (OM) and Field Emission Scanning Electron Microscopy (FE-SEM) were used to analyze the cross-sectional surfaces of the samples. Mechanical properties were evaluated through hardness and compression tests. The study reveals that the sample solution-treated at 520 °C exhibited the highest hardness and yield stress compared to those treated at 480 °C and 500 °C. The hardness of MDF samples increased from 82 HV to 165 HV as the aging temperature rose to 140 °C, where the highest hardness, flow stress, and yield strength were observed. At 190 °C for aging temperature, full recrystallization occurred, and at 240 °C and 290 °C, grain growth was observed, leading to a decrease in hardness, 128 HV and 97 HV, and yield strength, 505 MPa and 386 MPa, respectively. The results demonstrate that a solution treatment at 520 °C followed by artificial aging at 140 °C produces the best mechanical properties and microstructural characteristics in the AA2024 alloy, achieving the flow stress of 791 MPa and yield stress of 621 MPa.

## Introduction

 Pure aluminum and its alloys are essential in modern engineering and are the most commonly used non-ferrous alloys. Their popularity has grown significantly in recent years due to their lightweight nature, excellent corrosion resistance, favorable mechanical properties, and ease of machining and welding. Additionally, they are relatively cost-effective^1,2^. In order to replace steel with aluminum alloys, it is necessary to improve their physical and mechanical properties. The main approach to improve the strength of aluminum alloys is alloying and aging, but new methods based on straining and grain refining have been proposed^2,3^.

Common metal forming methods do not significantly increase strength, but Severe Plastic Deformation (SPD) processes have been developed to increase strength without causing the metal to break or crack. These methods can create a noticeable change in the microstructure of metals, and other effects on the aging behavior and refining of secondary phase particles. The principle of this method is to apply strain to the metal material without changing its external dimensions^4^. Several processes have been proposed for applying SPD to metal materials and achieving Ultra-Fine Grain (UFG) structures in laboratory and industrial dimensions, including pressing in the same angular channel, high-pressure twisting, cumulative rolling, limited deep pressing, and Multi-Directional Forging (MDF)^5–10^.

Annealing processes are crucial for optimizing the mechanical properties and microstructure of aluminum alloys. Among these processes, solution treatment annealing plays a key role, especially in aluminum alloys such as AA2024. This heat treatment involves subjecting the alloy to high temperatures, distinguishing it from other annealing methods. Solution treatment significantly influences both the mechanical properties and the microstructure of AA2024, preparing the alloy for the subsequent aging process and ensuring the desired performance characteristics are achieved^11–19^.

It is possible to improve microstructure, hardness, and yield strength after the deformation processes with various heat treatments, such as aging or artificial aging. At each stage of the artificial aging process, some of the stored energy is lost, and then the microstructure changes^20–23^. In 2024-aluminum alloy, all alloy components are almost dispersed in the so-called supersaturated matrix, and through the dynamic aging process, deposition starts with the formation of Cu-Mg atomic binary clusters^25–27^. As the aging temperature increases, fine deposits of S′/S ($\:{\text{A}\text{l}}_{2}$CuMg), which are the strengthening factor, are created; therefore, aluminum 2024 is placed in the category of Al-Cu-Mg alloys^24–26^.

The release of stored energy drives recovery and recrystallization, but the microstructure itself governs the development and growth of grain boundaries that transform into recrystallized sub-grains, as well as the orientation of the grains. Precipitation hardening in aluminum alloys occurs at relatively low temperatures, leading to the formation of intermetallic particles composed of the primary alloying elements, such as copper and magnesium^27,28^. The initial stage of the aging process involves accelerating the decomposition of the supersaturated solid solution, which ultimately results in the formation of coarse intermetallic particles^29,30^.

The mechanical properties of aluminum alloys are enhanced by precipitation hardening, which optimizes aging conditions and leads to further strengthening with continued aging. Aluminum alloy 2024 contains fine S′/S particles along with coarser intermetallic particles composed of Fe, Cu, and Mn. The combination of these fine and coarse particles influences precipitation, recrystallization, and grain refinement. Fine particles can restrict the movement of grain boundaries around coarser particles, thereby affecting recrystallization during aging. In alloys with coarse particles, particle-stimulated nucleation is a primary recrystallization mechanism during artificial aging. The presence of fine particles plays a crucial role in determining the critical diameter of coarse particles necessary for this phenomenon to occur^31–39^.

Given the significant influence of processing parameters on the mechanical properties and microstructure of the AA2024 alloy, identifying the optimal heat treatment temperatures is essential for achieving superior mechanical properties. This study aims to determine the most effective solution treatment and aging temperatures for the AA2024 aluminum alloy, improving its strength and microstructural integrity. Furthermore, the research investigates the complex interaction between precipitation strengthening and recovery mechanisms during deformation and subsequent artificial aging, providing critical insights for enhancing the mechanical properties of the alloy.

## Experimental material and procedure

In this research, an Al-Cu-Mg (AA2024) aluminum alloy was used, with its chemical composition, presented in Table 1. Samples measuring 10 mm × 10 mm × 15 mm were cut from the ingots for the multi-directional forging process (MDF).

**Table 1:** Chemical composition (mass fraction, %) of AA2024 aluminum alloy.

Al	Si	Fe	Cu	Mn	Mg	Cr	Zn
Base	0.11	0.34	3.86	0.44	1.23	0.01	0.29

**Fig. 1:**
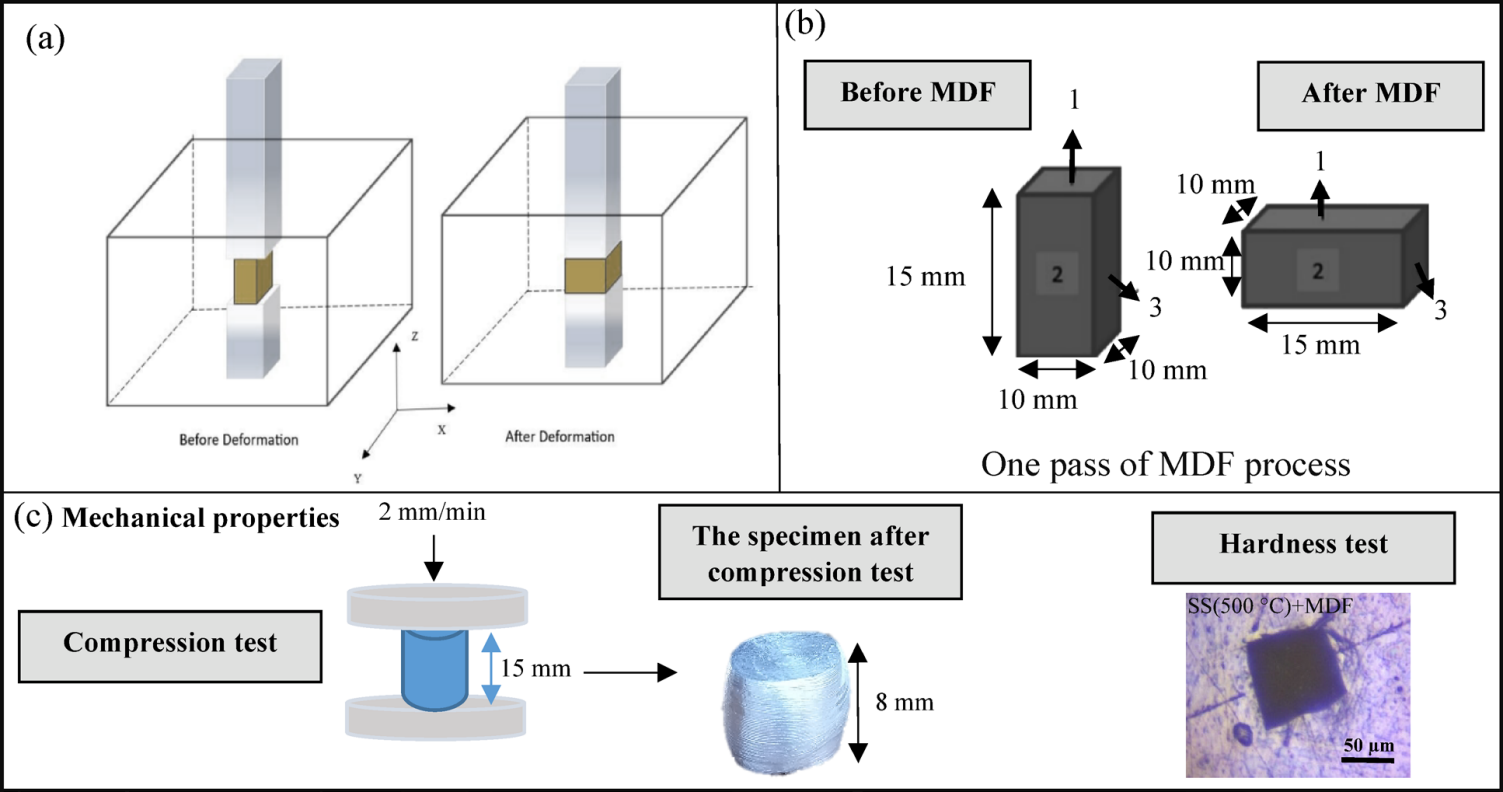
(**a**) Schematic of MDF process, (**b**) size of the sample before and after MDF process, (**c**) compression test, compressed sample and hardness indent.

The schematic of the MDF process is shown in Fig. 1(a). In this study, a MDF die with dimensions of 10 mm × 10 mm × 15 mm was used. The samples were pressed using a hydraulic press machine with the speed of 2 mm/min, as shown in Fig. 1(a). To minimize friction between the specimen and the die, Mo$\:{\text{S}}_{2}$ lubricant was applied. The size of the sample before and after MDF process are shown in the Fig. 1(b).

Based on Eq. 1 (where n represents the pass number, H the height, and W the width), the total strain after one MDF pass in this study was calculated to be 0.47.

1 $$\:{\upepsilon\:}=\frac{2\text{n}}{\sqrt{3}}\text{l}\text{n}\frac{\text{H}}{\text{W}}$$.

In the first stage of this study, the properties of AA2024 after the MDF process were compared to those of a non-MDFed sample. Both samples underwent solution treatment at 500 °C. In the second stage, the samples were subjected to solution treatment at three different temperatures: 480 °C, 500 °C, and 520 °C, for one hour in an electric oven, to identify the optimal solution temperature. The chosen temperatures (typically around 480 °C to 520 °C) are critical because they are high enough to facilitate the dissolution of $\:{\text{A}\text{l}}_{2}$Cu and $\:{\text{A}\text{l}}_{2}$CuMg phases into the aluminum matrix, creating a solid solution. The time at these temperatures is also crucial. Longer times can lead to over-aging or excessive grain growth, which can negatively impact mechanical properties^1,40^. Typically, solution treatment times range from 30 min to 2 h, depending on the thickness of the material and the specific alloy composition. Based on the explanations, 1 h for time and 480 °C, 500 °C, and 520 °C temperatures were selected for solution treatment. Afterward, a single pass of MDF was performed on the samples, followed by aging at 140 °C and cooling to room temperature. In the third stage, the solution treatment temperature was fixed at 500 °C. The samples were then subjected to a one-pass MDF process, followed by aging at temperatures of 100 °C, 140 °C, 190 °C, 240 °C, and 290 °C. The aging temperatures (commonly between 160 °C and 190 °C) are chosen to optimize the precipitation kinetics, promoting the formation of fine, coherent precipitates that strengthen the alloy without causing excessive grain-coarsening^35,40^. Considering the SPD process prior to aging, the selected aging temperatures were chosen to comprehensively evaluate the effect of aging at different temperatures after SPD.

**Fig. 2:**
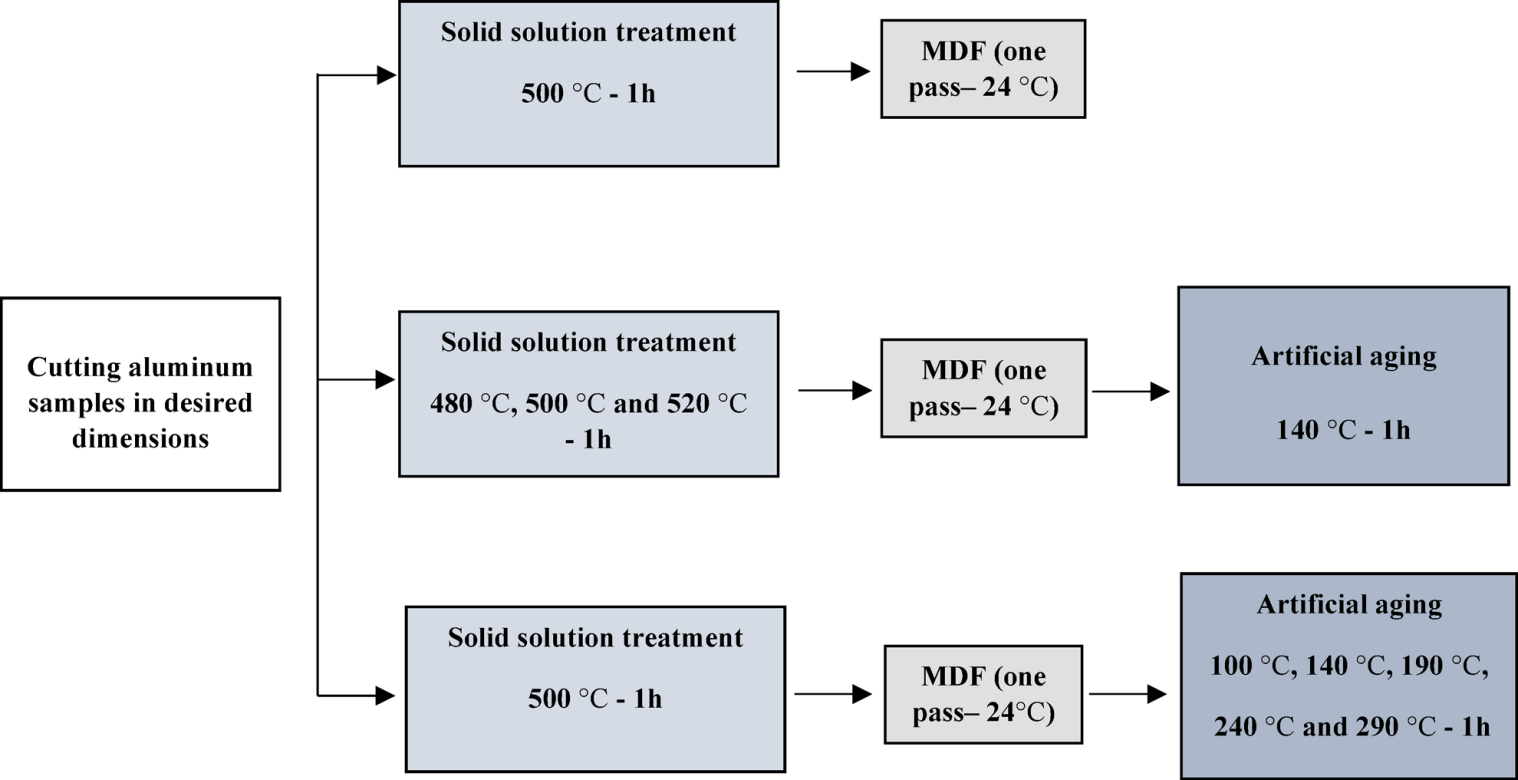
Schematic of research procedure.

The schematic of the research procedure is illustrated in Fig. 2. The compression test was conducted to evaluate the flow stress and yield stress of the samples. A height-to-diameter ratio of 1:1.5, deemed suitable for the compression test, was used. The test was performed at room temperature using the STM600 device, with a testing speed of 2 mm/min. The shape of sample after compression test is provided in Fig. 1(c).

The hardness of the samples was measured using a Vickers INSTRON WOLPERT hardness machine with a load of 20 kg/weight and a time of 20 s for each indentation, Fig. 1(c). The microstructure of the samples was analyzed using an optical microscope (OM) and a field emission scanning electron microscope (FE-SEM) equipped with energy-dispersive X-ray spectroscopy (EDS). The samples were prepared through standard grinding and polishing techniques, followed by electro-etching in a solution containing 3% HB$\:{\text{F}}_{4}$ (by volume) in distilled water. The electro-etching process was performed at a voltage of 20 volts for 2 to 3 min, depending on the sample. In order to asset the microstructure images, the grain size of samples was calculated through the linear intercept method within the IMAGE J software.

## Results and discussion

### Microstructure

#### MDF deformation of AA2024 alloy at room temperature

**Fig. 3:**
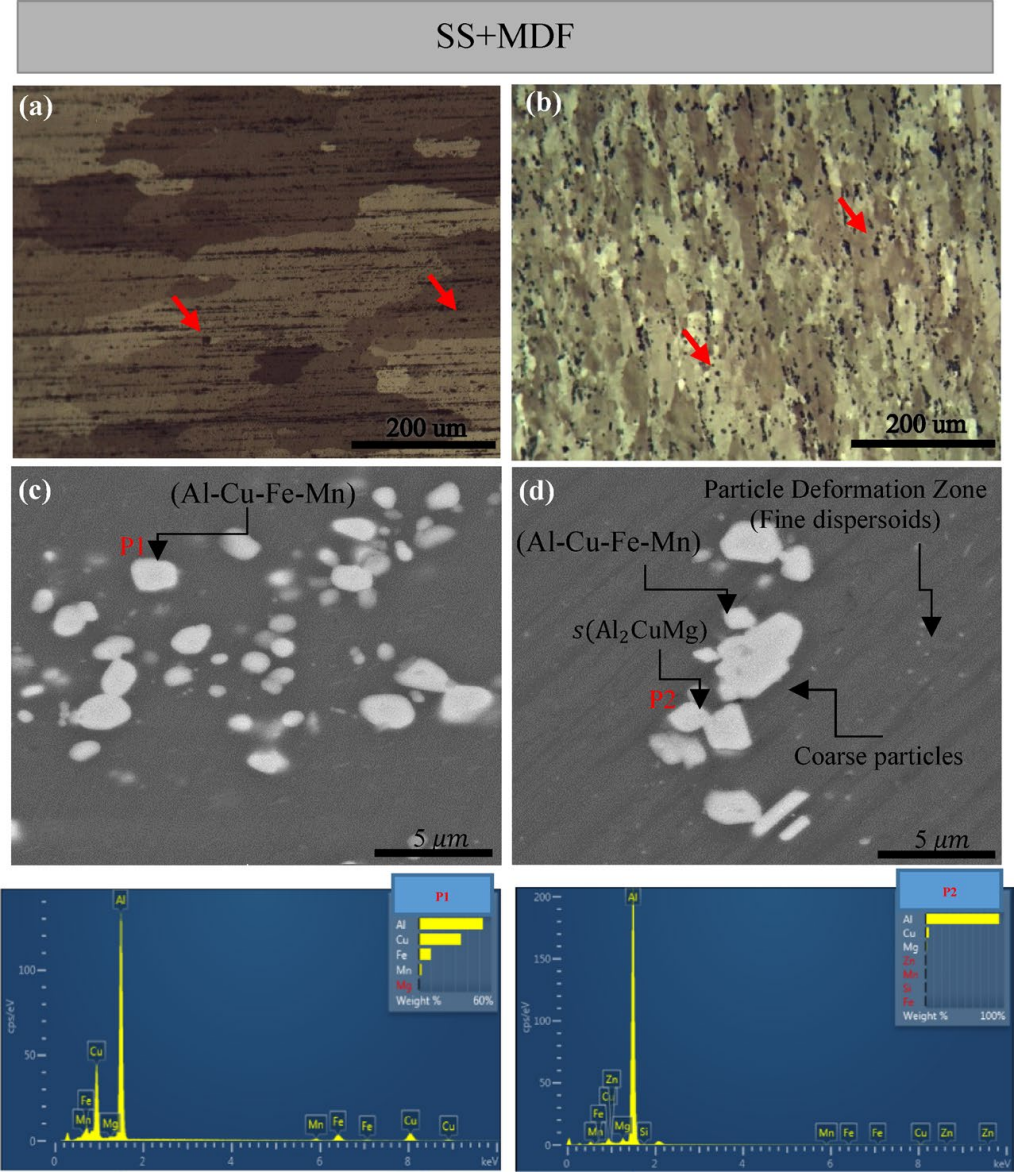
Optical microscope, FE-SEM images and EDS corresponding to the area marked in pictures (**c**,** d**), of solution treated AA2024 samples at 500 °C for 1 h, without MDF (**a**,** c**), with MDF (**b**,** d**).

In this section, the samples are first dissolved at 500 °C to investigate the effect of the MDF process on the AA2024 alloy. Figure 3(a, b) present optical micrographs of the microstructure for the samples dissolved at 500 °C, with and without the MDF process, respectively.

The microstructure of the solution sample, Fig. 3 (a), has coarse, homogenous, and coaxial grains with an average grain size of 120 μm. However, after processing MDF (Fig. 3 (b)), sub-borders and sub-grains have been created in the microstructure, which reduced the grain size to about 35 μm. The microstructure is observed to have a large number of shear bands parallel to the pressure direction. Two main factors, including initial grain size and dissolved atoms, are involved in the formation of shear bands^24,35^. It can be concluded that the occurrence of shear bands is more in the microstructure of MDF sample, Fig. 3(b). In addition, the precipitants are mentioned with red alignments in the images.

AA2024, a precipitation-hardenable alloy, in its solution state, when subjected to multidirectional forging, dynamic precipitation of Cu-Mg atomic binary clusters (GPB) occurs. In fact, dynamic deposition causes premature aging before artificial aging and is closely related to the phenomenon of dynamic aging. Due to the high density of dislocations and the increase in the effect of the atomic diffusion coefficient in the microstructure, clusters of dissolved atoms are formed under the dislocations, and when the dislocations are released from their tensile force, the remaining clusters behind the dislocations can lead to the germination of particles^36-44^.

FE-SEM and EDS results, Fig. 3 (c, d) illustrate the presence of deposits and coarse compounds in the microstructure, especially in supersaturated samples, the presence of $\:{\text{A}\text{l}}_{6}$(Cu-Fe-Mn) particles that do not dissolve during dissolution and deformation is observed in Fig. 3(c), marked by P1. Also, a part of the small S grains, whose existence was confirmed in Fig. 3 (P2), appeared next to the coarse grains and are distributed inhomogeneously in the microstructure, which is caused by the non-uniformity of the strain distribution. The existence of this heterogeneity in the microstructure, as mentioned, is on the one hand due to the initial microstructure and on the other hand due to the heterogeneity of the multi-directional forging process.

Multidirectional forging has also accelerated the solution of dissolved atoms by producing moving boundaries. The solubility of atoms in a moving boundary is much higher than that of a stationary boundary due to the large free volume. Also, the reason for the increased density of shear bands in the supersaturated deformation sample can be attributed to the higher volume fraction of GPB regions in the multidirectional forging supersaturated sample. Kang et al.^45^ also observed this dynamic deposition in 2024 aluminum alloy after applying two deformation passes in an equal-angle channel at room temperature, but in their research, dynamic deposition of $\:{\text{A}\text{l}}_{2}$Cu or θ phase was observed. Huang et al.^46^ did not observe any semi-stable phase such as GPB in Al alloy with 4 wt% Cu with severe plastic deformation at strain 10 and aged at room temperature. Considering a critical strain, if the amount of strain given in MDF is less than this value, GPB regions or Cu-Mg binary clusters are formed, and in the present research, semi-stable phases are formed at this stage, which causes that dynamic aging occurs before artificial aging. The applied strain does not completely affect the microstructure due to the grain refinement process in semi-stable phases because a fraction of Cu-Mg binary clusters is not dynamically formed. The amount of semi-stable particle produced, which is a suitable place for the creation of small S’/S deposits ($\:{\text{A}\text{l}}_{2}$CuMg), is not enough to produce a large number of S’/S deposits.

#### Effects of SST at various temperatures before MDF and aging

This section aims to identify the optimal solution treatment temperature for improving the mechanical properties and microstructure of AA2024. To achieve this, samples were heat-treated at 480 °C, 500 °C, and 520 °C for one hour prior to the MDF process. Subsequently, all samples were aged at a fixed temperature of 140 °C for one hour.

**Fig. 4:**
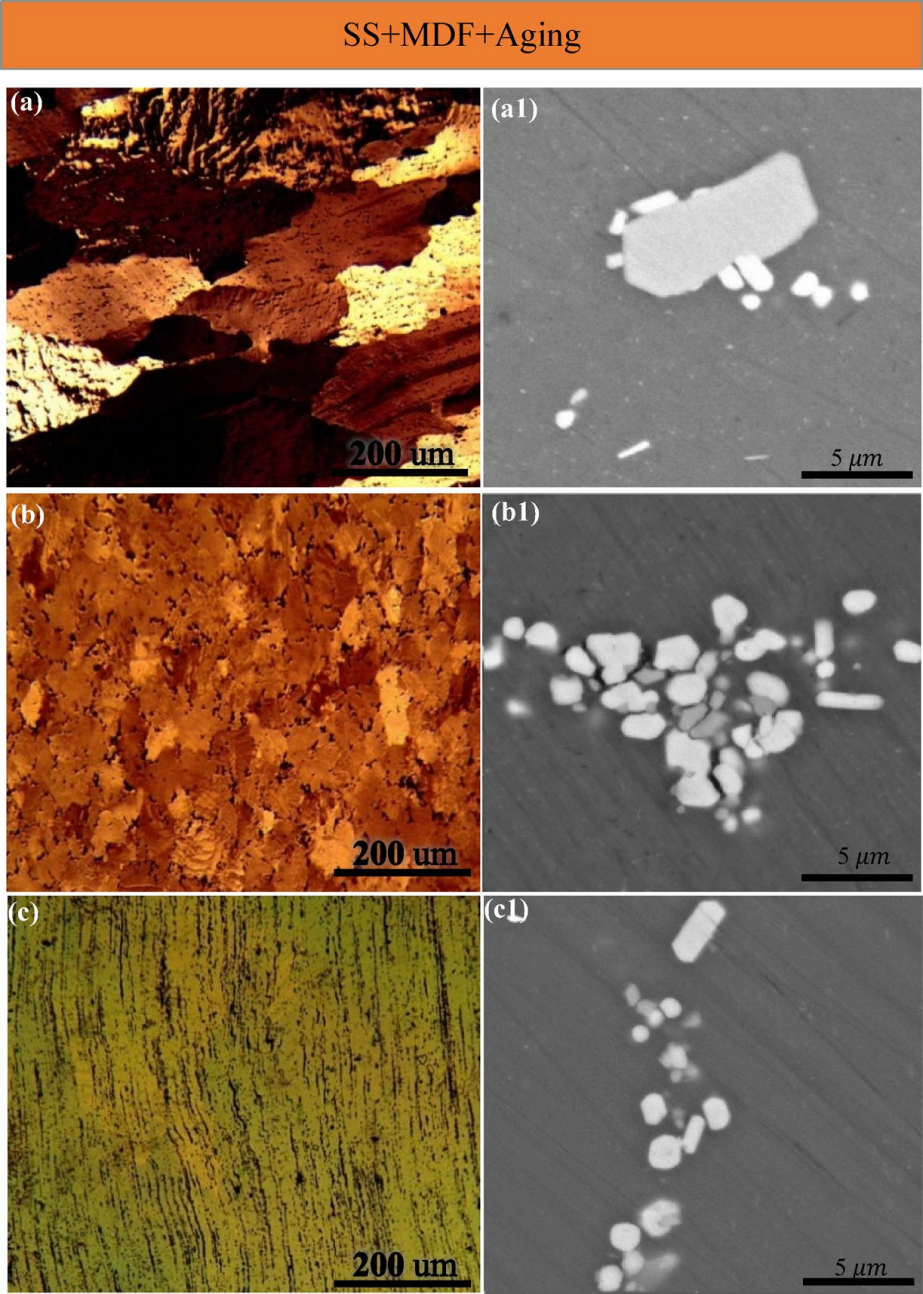
Optical microscope and FE-SEM images of solution treated AA2024 samples at: (**a**), and (**a1**) 480 ℃, (**b**), and (**b1**) 500 ℃, and (**c**), and (**c1**) 520 ℃ for 1 h Post-MDF (single pass) and aged at 140 ℃ for 1 h.

Figure 4 presents the microstructure of the AA2024 samples solution-treated at 480 °C, 500 °C, and 520 °C for one hour, followed by a single-pass MDF process at room temperature and subsequent aging at 140 °C for one hour.

According to zener locking formula (Eq. 2) If the distance between the fine-phase particles (λ) is much smaller than the diameter of sub-grains (2R) (λ<<2R), the static recovery phenomenon will be affected by these particles. In many cases, a final limit for the sub-grain diameter is predicted, whose relationship is expressed as follows^47^:

$$\:\:\:D=2R=\frac{2\alpha\:r}{3{F}_{V}}\:\:\:\:\:\:\:\:\:\:\:\:\:\:\:\:\:\:\:\:\:\:\:\:\:\:\:\:\:\:\:\:\:\:\:\:\:\:\:\:\:\:\:\:\:\:\:\:\:\:\:\:\:\:\:\:\:\:\:\:\:\:\:\:\:\:\:\:\:\:\:\:\:\:\:\:\:\:\:\:\:\:\:\:\:\:\:\:\:\:\:\:\:\:\:\:\:\:\:\:\:\:\:\:\:\:\:\:\:\:\:\:\:\:\:\:\:\:\:\:\:\:\:\:\:\:\:\:\:\:\:\:\:\:\:\:\:\:\:\:\:\:\:\:\:\:\:\:\:\:\:\:\:\:\:\:\:\:\:\:\:\:\:\:\:\:\:\:\:\:\left(2\right)$$

In this relationship, α is a constant number (~ 1.5), FV and r are the volume fraction and radius of particle S, respectively. According to the above relation, the final diameter of the sub-grain has an inverse relationship with the volume fraction of particle S (D∝F_V_^−1^) and is directly proportional to the size of the particles.

Considering Fig. 4(a, b, and c), with an increase in solution treatment temperature, grain size decreases which is related to variation in particle size and distribution. The grain sizes of these samples are 160 μm, 77 μm, and 74 μm, respectively. In addition, grain size and dissolved atoms lead to create the shear bands in the microstructure of dissolved sample at 520 °C.

Analyzing FE-SEM pictures, Fig. 4(a1, b1, and c1), particles are very coarse at 480 °C. According to the Eq. 2, the weak Zener locking effect of these particles is due to the fact that the diameter of these particles is larger than the critical diameter, and sub-grain boundaries grow easily.

In addition, the particles become finer due to increasing the temperature up to 500 °C. However, particles dissolved at a temperature of 520 °C, because there is a suitable and sufficient temperature and opportunity for dissolution, most of the semi-stable particles of the matrix are dissolved, a supersaturated solid solution is obtained. Moreover, the structure becomes more unstable, along with coarse particles that have a more suitable diameter and a more uniform distribution. found, there are small, scattered, and homogenous partial stable deposits of S due to aging heat treatment.

#### Effects of aging at various temperatures after SST and MDF deformation

**Fig. 5:**
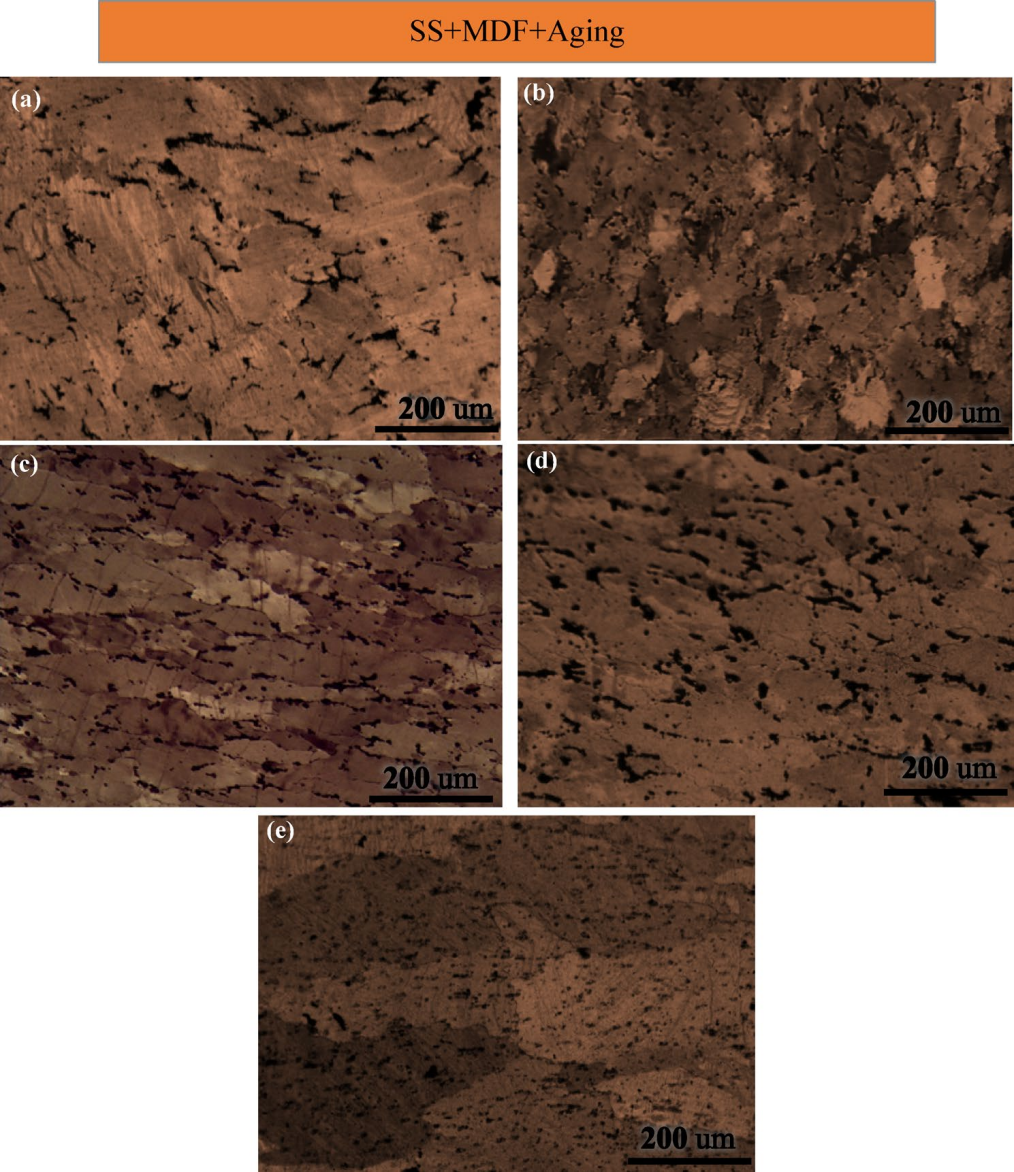
Optical microscope images of aluminum alloy 2024 dissolved samples at 500 ℃ for 1 h, post-MDF (single pass) and aged at (**a**) 100 ℃, (**b**) 140 ℃, (**c**) 190 ℃, (**d**) 240 ℃, (**e**) 290 ℃ for 1 h.

Figure 5 shows the microstructure of the samples dissolved at 500 °C for 1 h, subjected to post-MDF, and then aged at (a) 100 °C, (b) 140 °C, (c) 190 °C, (d) 240 °C, and (e) 290 °C.

The grain size is smaller in the sample aged at 140 °C, 77 μm, compared to the one aged at 100 °C, 106 μm, indicating that recrystallization began at 140 °C. The size of the recrystallized grains can be influenced by several factors affecting the nucleation and growth processes. Factors such as the amount of strain, the presence of insoluble primary deposits, and the temperatures during solution treatment and aging all play a role. As the solution treatment temperature, applied strain, or aging temperature increase, the effects of these factors diminish due to the higher driving force provided by the elevated starting temperature of recrystallization and grain growth.

Several factors suggest that the sample underwent the recovery phenomenon during the final stages of the one-hour at 100 °C. The heating process promotes the rearrangement of high-density dislocations induced by MDF into a more organized structure. This rearrangement leads to the formation of numerous sub-boundaries, significantly enhancing the likelihood of rapid recovery. In addition, aluminum alloy possesses a high stacking fault energy (SFE), enabling dislocations to climb and slide with ease. While the primary grain boundaries exhibited minimal migration, the sub-grains increased in size. As the temperature rose, the grains became more homogenized and better aligned. Recovery also depends on the concentration of dissolved atoms. Notably, the dynamic deposition of GPB regions increases the recovery rate by reducing the number of dissolved atoms and enhancing the mobility of dislocations. At this stage, some semi-stable GPB deposits dissolve, and the recovery phenomenon is observed through the dissolution of GPB regions in the aged alloy at 100 °C. Moreover, at lower temperatures, the formation and growth of semi-stable particles are more pronounced compared to stable particles. Specifically, the volume fraction of S precipitates, formed when the sample is aged at 100 °C for 1 h, is greater than that observed in the sample that is supersaturated after undergoing MDF. Most of the S deposits have a very small size on the sub-boundaries, which do not have a high locking effect at the beginning, and at the end of one hour, the diameter of the deposits is increasing, which causes them to exert more Zener pressure than the MDF case. The probability of recrystallization with the nucleation mechanism stimulated by particles decreases at the beginning of aging at this temperature, and as a result, it increases the probability of the recovery phenomenon.

In the sample aged at 140 °C, the coarse particles exceed the critical size, facilitating PSN (Particle Stimulated Nucleation) germination in their vicinity. During deformation, these coarse particles alter the strain direction around them. The high stored energy in the regions surrounding the deformed particles accelerates the germination of new grains during artificial aging. In this study of an aluminum alloy containing S deposits, the critical diameter of coarse intermetallic particles (​$\:{d}_{\text{c\:}}$) is influenced by the grain boundary surface energy (​$\:{{\upgamma\:}}_{\text{b}}$) and the Zener locking pressure exerted on the grain boundaries by the S deposits^47^:

3 $$\:\:\:\:\:\:\:\:\:{d}_{\text{c\:}}=\frac{{2\gamma\:}_{b}}{3{(P}_{D}-{P}_{z})}\:\:\:\:\:\:\:\:\:\:\:\:\:\:\:\:\:\:\:\:\:\:\:\:\:\:\:\:\:\:\:\:\:\:\:\:\:\:\:\:\:\:\:\:\:\:\:\:\:\:\:\:\:\:\:\:\:\:\:\:\:\:\:\:\:\:\:\:\:\:\:\:\:\:\:\:\:\:\:\:\:\:\:\:\:\:\:\:\:\:\:\:\:\:\:\:\:\:\:\:\:\:\:\:\:\:\:$$.

As the volume fraction of the particles increased, some of the S′/S ($\:{\text{A}\text{l}}_{2}$CuMg) precipitates dissolved due to the elevated temperature, promoting further precipitation. Simultaneously, part of the coarse intermetallic, quasi-stable, and stable S phases in the sample became coarser during the 1-hour at 140 °C. This coarsening process reduced their volume fraction, leading to a decrease in Zener locking effect. Consequently, recrystallization occurred to a greater extent by the end of the 1-hour duration. Therefore, in addition to deformation, temperature and time also contribute to numerous inhomogeneities^48^. Due to the large orientation gradient (Ω), these regions are considered prone sites for the nucleation of recrystallized grains^47^. The recrystallization process, characterized by the gradual transformation of low-angle grain boundaries (LAGBs) into high-angle grain boundaries (HAGBs), requires that small-angle boundaries do not migrate too quickly, as they can be destroyed if they move excessively^49^. At the temperature of 140 °C, the few large-angle boundaries are attributed to the occurrence of the PSN phenomenon.

The fundamental difference between recovery and recrystallization lies in the relative fractions of small and large-angle grain boundaries^50^. The presence of an orientation gradient is critical for recrystallization nucleation, as areas with large orientation gradients store more energy due to the higher density of LAG boundaries^49^. It is important to note that recovery, which is a precursor to recrystallization, occurs very rapidly in regions with high orientation gradients. The smallest diameter to which a secondary grain must grow ($\:{D}_{\text{lim\:}}$) to form a HAG boundary is inversely related to the orientation gradient. In other words, the criterion for recrystallization nucleation can be approximated as follows^47^:

4 $$\:{\text{D}}_{\text{lim\:}}\ge\:\frac{{\theta\:}_{m}}{{\Omega\:}}$$.

As shown in Fig. 5(c), the grain size of the sample subjected to artificial aging at 190 °C, 75 μm, is smaller than that of the sample aged at 140 °C. This difference is attributed to the increased volume fraction and growth of S deposits in the sample aged at 190 °C. The enhanced growth reduces the Zener pressure acting on the sub-boundaries, facilitating complete recrystallization. By the end of the one-hour, a microstructure comprising coaxial and quasi-coaxial grains is formed. During recrystallization, the density of crystal defects decreases, and this phenomenon interacts with the dissolution of $\:{\text{A}\text{l}}_{2}$CuMg S’/S deposits.

The grain size increased at the aging temperature of 240 °C to about 120 μm. When the temperature exceeds the optimum for recrystallization, grain size increases, resulting in coarser grains visible in the microstructure. Particle coarsening occurs primarily through grain boundary penetration, affecting only particles located within the grain boundaries, while other particles coarsen at a slower rate. The growth of S particles reduces the intensity of their locking effect. Additionally, the microstructure of the sample aged at 290 °C reveals even larger grains compared to the sample aged at 240 °C with an average grain size of 170 μm. This can be attributed to the growth and partial dissolution of particles at the higher temperature.

**Fig. 6:**
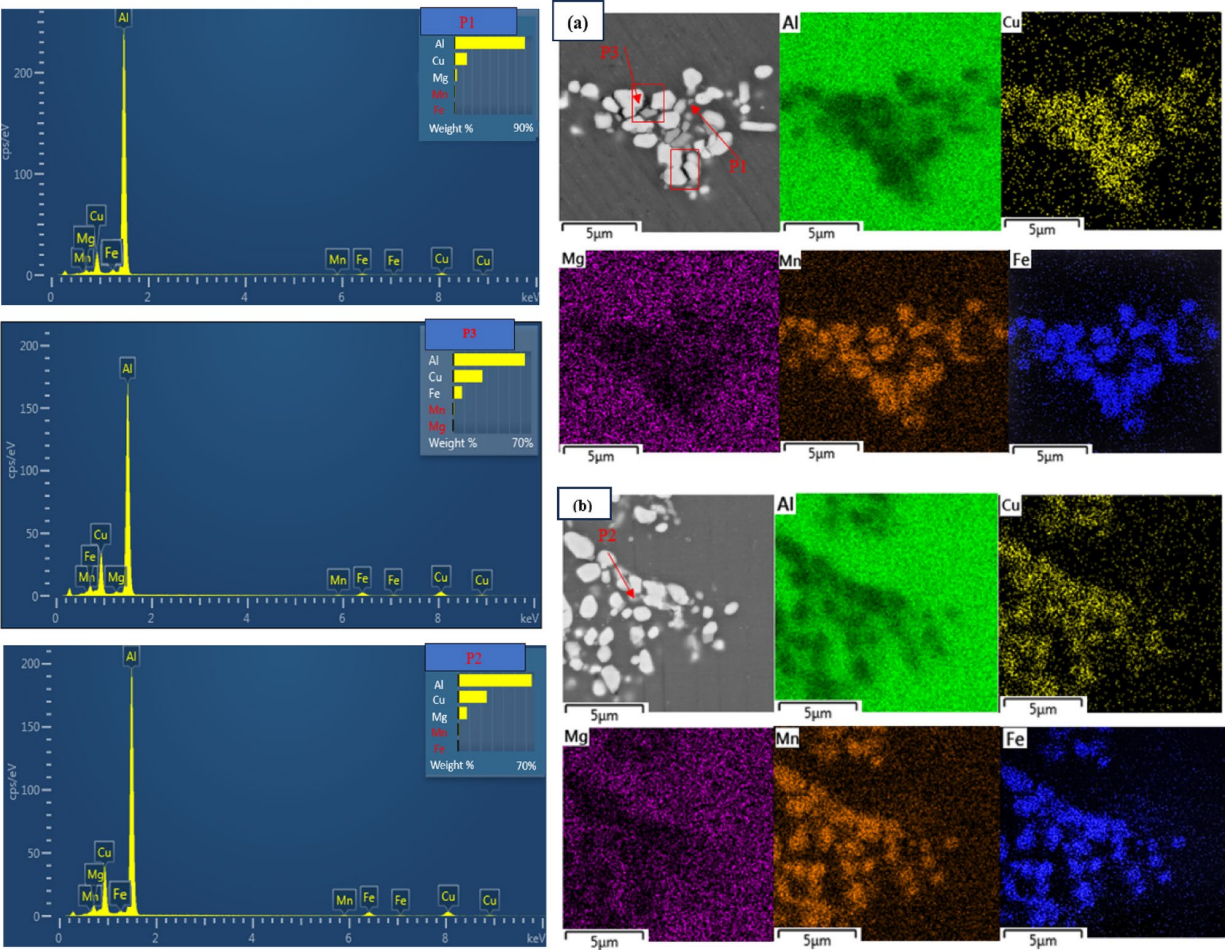
Comparison of SEM images and elemental analysis maps of 2024 aluminum alloy solution samples at 500 °C for 1 h, after MDF (single pass) and aged at (**a**) 100 °C, (**b**) 140 °C for 1 h and EDS corresponding to the area marked in figure (**a**,** b**).

Figure 6 shows the FE-SEM image and elemental analysis maps of the 2024 aluminum alloy sample aged at 500 °C for 1 h, after MDF (single pass), aged at 100 °C and 140 °C for 1 h.

As illustrated in Fig. 6 (P1, P2), the relatively high concentrations of Mg and Cu elements in the specified regions indicate the formation of more stable ($\:{\text{A}\text{l}}_{2}$CuMg) S deposits in the matrix. These deposits appear slightly coarser in structure. The sufficient volume fraction and favorable growth of S or $\:{\text{A}\text{l}}_{2}$CuMg deposits observed in the 140 °C sample, compared to the 100 °C sample, suggest that recrystallization begins to occur at this stage. Additionally, the co-deposition of S particles, accompanied by a significant fraction of small-angle boundaries in the sample aged at 100 °C, may indicate the onset of recovery. S deposits are located on boundaries that can be classified as sub-boundaries due to the recovery process. As shown in Fig. 6 (P2), during artificial aging up to 140 °C, the volume fraction of S’/S ($\:{\text{A}\text{l}}_{2}$CuMg) deposits increases, and the rate of Zener locking rises with the driving force. This increase is attributed to the reduced recovery at higher temperatures, which raises the critical diameter of coarse particles required for PSN to occur. Around these coarse particles, small recrystallized grains with high-angle grain boundaries are observed, formed through the PSN mechanism.

As evident from the elemental maps in Fig. 6, higher temperatures lead to a more uniform distribution of manganese, iron, and copper in the matrix. Additionally, magnesium is distributed within the deposits as stable and semi-stable phases form. The phase particles are copper-rich, and their formation during deposition depletes the copper content in the surrounding matrix^51^.

According to Fig. 6(a, P3), the elemental map, and the corresponding analysis, areas free of precipitation are observed during artificial aging up to 100 °C. These areas, visible as parallel dark lines in the figure, are regions where coarse intermetallic particles such as $\:{\text{A}\text{l}}_{7}{\text{C}\text{u}}_{2}$Fe have formed. The presence of $\:{\text{A}\text{l}}_{7}{\text{C}\text{u}}_{2}$Fe intermetallic particles is confirmed through EDS chemical analysis and elemental mapping.

**Fig. 7:**
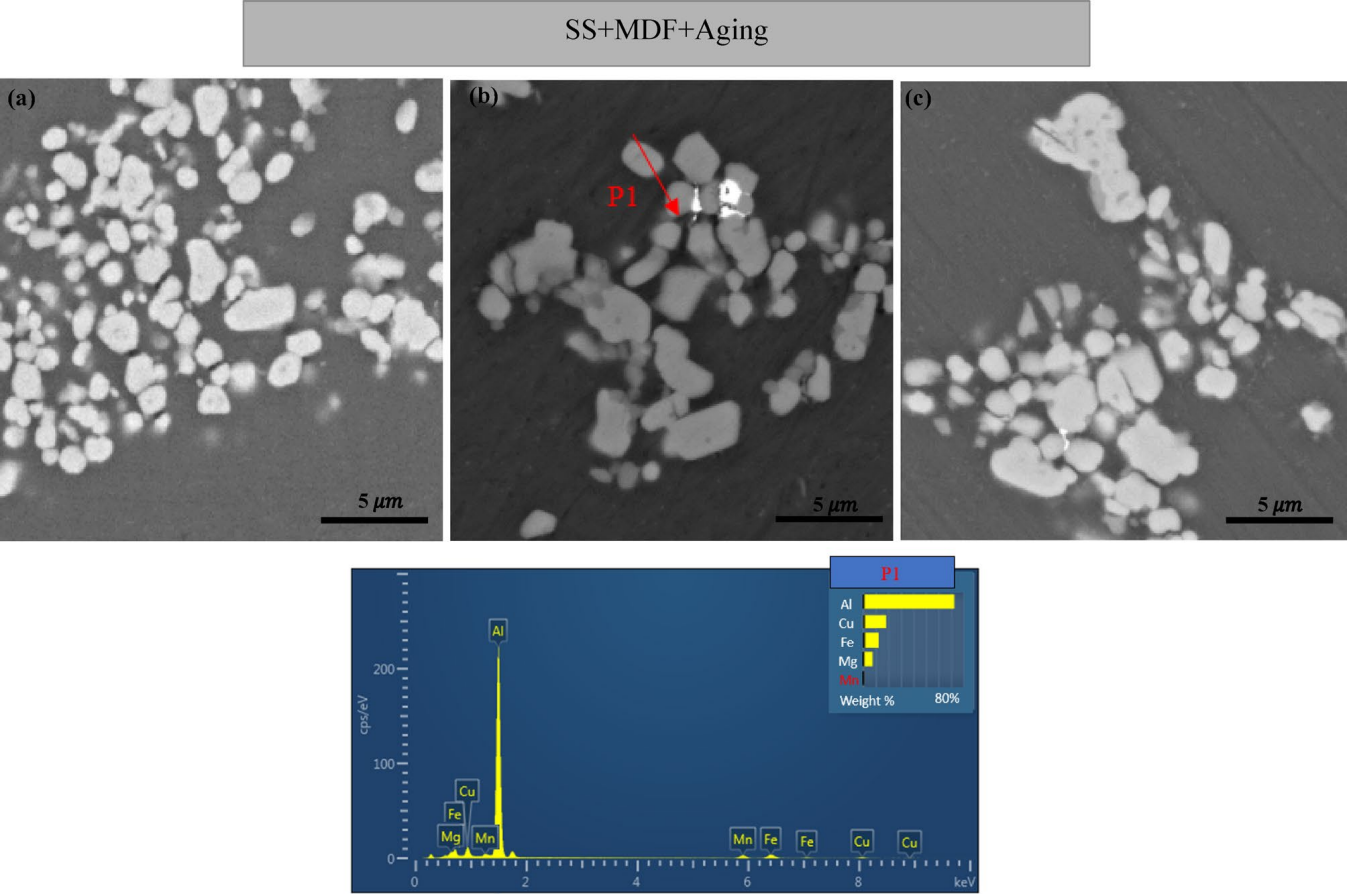
FE-SEM images of aluminum alloy 2024 dissolved samples at 500 ℃ for 1 h, post-MDF (single pass) and aged at (**a**) 190 ℃, (**b**) 240 ℃, (**c**) 290 ℃ for 1 h and EDS Corresponding to the area marked in Figure (**b**).

In Fig. 7, FE-SEM images of samples aged at three different temperatures are shown, demonstrating that deposit growth increases with aging temperatures from 190 °C to 290 °C. The sample aged at 190 °C (Fig. 7(a)) exhibits a higher volume fraction of stable deposits compared to the sample aged at 140 °C. However, with the increase in temperature, some of the S deposits dissolve, leading to a reduction in their volume fraction. Additionally, the partial dissolution of both semi-stable and stable deposits continues, resulting in a slight network instability and ultimately precipitation.

Simultaneously, the growth of a significant number of deposits occurs. Recrystallization grains form around coarse deposits, primarily composed of insoluble $\:{\text{A}\text{l}}_{7}{\text{C}\text{u}}_{2}$Fe and $\:{\text{A}\text{l}}_{20}{\text{C}\text{u}}_{2}{\text{M}\text{n}}_{3}$, due to the elevated temperatures.

As shown in Fig. 7(b), the particles in the aged sample coarsened significantly at 240 °C, and the Zener locking effect was almost eliminated. Furthermore, in Fig. 7(c), the particles grew even larger and became coarser than those in the samples aged at 190 °C and 240 °C. Along with this growth, there was also a partial dissolution of the S’/S volume fraction.

**Fig. 8:**
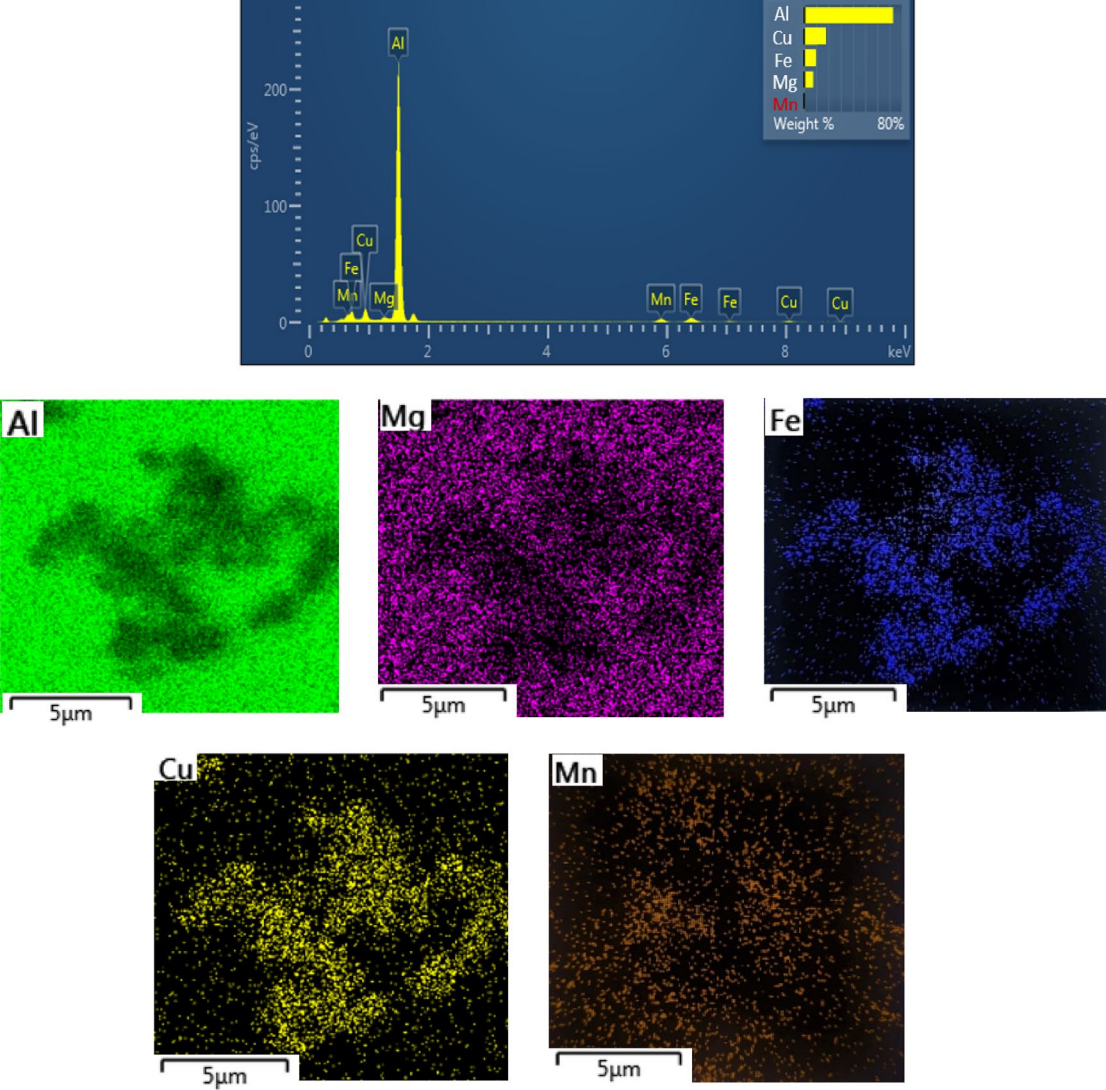
EDS and elemental analysis maps of intermetallic particles of 2024 aluminum alloy solution samples at 500℃ for 1 h, post-MDF (single pass) and aged at 240℃ for 1 h.

Figure 8 presents the elemental analysis maps of intermetallic particles in solution-treated aluminum alloy 2024 samples, processed at 500 °C for 1 h following MDF (single pass) and aged at 240 °C for 1 h. The corresponding EDS analysis for the specified area is also shown. With increasing temperature, while the deposits and intermetallic compounds remain insoluble, their growth is evident. Moreover, the distribution of their constituent elements within the microstructure becomes more uniform, as confirmed by Fig. 8 and the EDS analysis.

### Changes in mechanical properties by SST, MDF, and aging

**Fig. 9:**
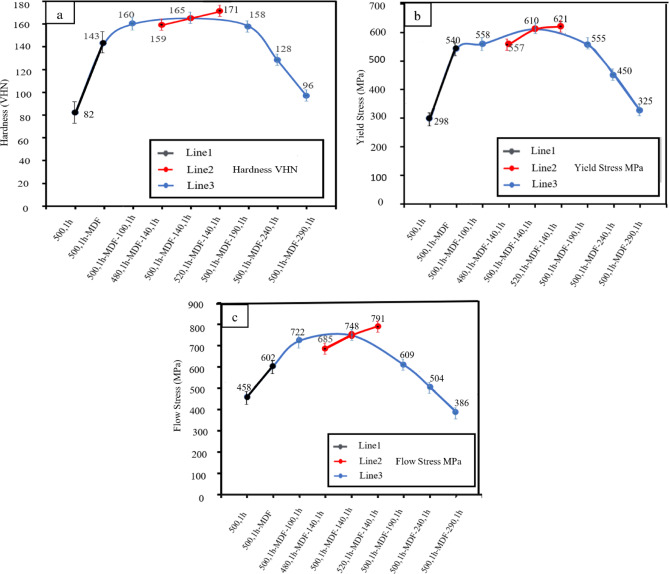
Variation in (**a**) hardness, (**b**) yield stress, (**c**) flow stress.

Figure 9(a–c) illustrates the changes in hardness, flow stress, and yield stress of the dissolved and aged samples across various temperatures.

#### MDF deformation of AA2024 alloy at room temperature

Figure 9(a), Line 1, shows the hardness, yield stress, and flow stress of the samples dissolved at 500 °C, both before and after the MDF process. As discussed in previous section, the hardness increased from 82 to 143 Vickers after the MDF process. This increase in hardness is attributed to the enhanced penetration, which results from the higher dislocation density, the formation of sub-boundaries, the development of cell walls, and dynamic precipitation.

Additionally, data extracted from the compression tests showed that the yield stress, Fig. 9(b), Line 1, increased from 298 MPa to 540 MPa, while the flow stress, Fig. 9(c), Line 1, rose from 458 MPa to 602 MPa for the samples before and after the MDF process, respectively.

As shown in Fig. 9(b, c), Line 1, the MDF process increases the flow and yield stress. This increase in stresses is attributed to the dynamic deposition of GPB regions or Cu-Mg atomic binary clusters and the accumulation of dislocations. It should be remembered that the GPB regions of fine particles can be cut. The interaction of atomic displacements caused by dislocations and the strain field around the precipitates leads to an increase in strength in the alloy^52^. In addition, by performing MDF, the sample becomes fine-grained, and according to the Hall-Patch equation, reducing the grain size also increases the strength^13^.

#### Effects of SST at various temperatures before MDF and aging

Figure 9(a) Line 2, shows the hardness variation of dissolved samples at 480 °C, 500 °C, and 520 °C for 1 h before MDF and aging at 140 °C, prove that with the increase in temperature, the hardness values reached 159 Vickers, 165 Vickers, and 171 Vickers, respectively. At first, it is an example of a strengthening solution with a solid solution mechanism. The highest degree of hardness is obtained at a solution temperature of 520 °C for 1 h. Due to the dissolution of the particles to the extent that their size (according to Eq. 2) has the greatest Zener effect on the sub-boundaries.

Figure 9 (b, c), Line 2, shows the changes in the flow stress and yield strength of the samples dissolved at temperatures of 480 °C, 500 °C, and 520 °C, respectively. The sample at 520 °C shows the highest amount of flow stress,791 MPa, and yield stress, 621 MPa, Because, as mentioned, the dissolution of particles has been done to the extent that it has the greatest effect on zener locking in the sub-boundaries. Therefore, it is concluded that the most optimal temperature and time for increasing flow stress and yield stress is 520 °C for 1 h.

#### Effects of aging at various temperatures after SST and MDF deformation

Figure 9(a), Line 3, illustrates the hardness of samples after artificial aging. By combining the heat treatment of aging on the supersaturated samples of multidirectional forging, even higher hardness can be reached, and the structure becomes homogeneous faster.

In artificial aging at 100 °C, the hardness value is 160 Vickers, which is lower than the hardness of the sample aged at 140 °C, which is 165 Vickers. For the sample aged at 100 °C, the recovery rate increases due to the dynamic precipitation of GPB and S phases, accompanied by a decrease in the number of dissolved atoms and an increase in the mobility of dislocations in the matrix. The increase in hardness in the sample aged at 140 °C is attributed to the precipitation of ($\:{\text{A}\text{L}}_{2}$CuMg) S′/S and PSN phases, which enhances Zener locking and strengthens the material. Recrystallization occurring after aging at 140 °C results in the formation of new grains with lower dislocation density and a finer microstructure compared to the sample aged at 100 °C. This grain refinement contributes to the observed increase in hardness.

By increasing the aging temperature to 190 °C, due to the completion of recrystallization, the reduction of the density of crystal defects (dislocations, etc.), and the reduction of internal stresses, the hardness decreased to 158 Vickers, which confirms the discussion of microstructural investigation. For example, despite the further decrease in grain size, there is also a decrease in the density of dislocations, which has caused it to experience a decrease in hardness, so that a competitive phenomenon of decrease in hardness due to a decrease in the density of dislocations and an increase in hardness due to the falling of grains is observed, and the reduction of dislocations has a more dominant effect at this temperature^52^.

The hardness of the samples aged at 240 °C and 290 °C for 1 h decreased to 128 Vickers and 97 Vickers, respectively. As the aging temperature increases to 240 °C, fine precipitates are partially dissolved, while insoluble intermetallic particles and stable precipitates grow to a size where they can no longer effectively lock the grain boundaries. This results in grain coarsening, which continues as the temperature rises to 290 °C, leading to a reduction in mechanical properties.

Figure 9(b, c), Line 3, shows the flow and yield stress for samples after artificial aging. After aging at 100 °C for 1 h, the yield stress and flow stress increase to 558 MPa and 722 MPa, respectively, and the reason for this increase can be attributed to the volume fraction of semi-stable and stable particles. Despite the fact that little recovery and dissolution of semi-stable particles is being done, the increase in volume fraction of particles has a dominant effect on this issue.

With an increase in aging temperature, the sample aged at 140 °C for 1 h shows an increase in yield stress and flow stress to 610 MPa and 748 MPa, respectively. This increase in stresses is attributed to the deposition of $\:{\text{A}\text{l}}_{2}$CuMg in the S′/S phase. The 140 °C aging temperature is the most optimal for achieving the highest flow and yield stress among the aging temperatures studied. At this temperature, the sample exhibits a non-uniform grain structure with both small and medium-sized grains. However, there is significant precipitation of fine particles due to the instability and supersaturation of the matrix, caused by the dissolution of quasi-stable particles and partial dissolution of the S′/S phase. This precipitation process, driven by severe plastic deformation and the increased temperature, occurs at a higher rate than the dissolution of particles, leading to a more pronounced effect. As a result, the fine-grained structure exerts a stronger influence on the material’s properties than the reduction in dislocation density.

In the next step, by increasing the aging temperature to 190 °C for 1 h, the particles become smaller and more dispersed, promoting PSN (particle-stimulated nucleation) in more regions. These well-distributed particles help stimulate grain nucleation^52^. However, as the particles have grown significantly, their ability to enhance Zener locking is reduced, which contributes to a more complete recrystallization process. As a result, the yield stress and flow stress decrease to 555 MPa and 609 MPa, respectively.

In the subsequent stages, with further increases in the aging temperature to 240 °C and 290 °C, the yield stress and flow stress decrease to 450 MPa and 504 MPa, respectively, at 240 °C, and to 325 MPa and 386 MPa at 290 °C. This reduction can be attributed to grain growth, which weakens the material’s mechanical properties.

Overall, the sample that was supersaturated before the MDF process at 520 °C and then subjected to artificial aging at 140 °C exhibits the most optimal mechanical properties for the AA2024 aluminum alloy.

## Conclusions

In this research, the effects of the artificial aging process and primary solution treatment at different temperatures on aluminum alloy 2024 were investigated, and the following results were obtained:

1. The solution treatment at 520 °C was identified as the optimal solution treatment condition, resulting the hardness of 171 Vickers, flow stress of 791 MPa, and yield stress of 621 MPa, after MDF and artificial aging at 140 °C.
2. Aging supersaturated aluminum alloy 2024 at 100 °C for one hour after MDF induces Cu-Mg cluster dissolution and recovery, with minimal $\:{\text{A}\text{l}}_{2}$CuMg (Sʹ/S) precipitation. S deposits, located within grains and sub-boundaries, lock sub-boundaries, delaying recrystallization until the temperatures reach between 140 and 190 °C.
3. Aging at 140 °C increases the volume fraction of Sʹ/S precipitates while dissolving semi-stable and stable phases. This process enhances hardness and microstructure. The temperature also promotes partial Sʹ/S phase dissolution, improving supersaturation and aging capabilities. As a result, this temperature is the most optimal temperature for artificial aging.
4. Aging at 140 °C and 190 °C promotes recrystallization around coarse particles. Increasing the temperature reduces hardness and strength due to complete recrystallization in the deformed supersaturated sample.

## Data Availability

The data that support the findings of this study are available from the corresponding author upon request.
